# CNKSR2 expression is correlated with immune infiltrates in Cervical Cancer as a favorable prognostic factor

**DOI:** 10.7150/jca.87622

**Published:** 2024-01-01

**Authors:** Guangxiao Li, Qiaoqiao Chen, Shihao Hong, Xiaoli Yang, Xiaoling Liang, Jianhua Yang

**Affiliations:** 1 Department of Obstetrics and Gynecology, Sir Run Run Shaw Hospital, School of Medicine, Zhejiang University, Hangzhou, 310020, China.; 2 Center for Reproductive Medicine, Drum Tower Clinic Medical College of Nanjing Medical University, Nanjing, Jiangsu, China.; 3 Key Laboratory of Reproductive Dysfunction Management of Zhejiang Province Assisted Reproduction Unit, Sir Run Run Shaw Hospital, Zhejiang University School of Medicine, Hangzhou, 310020, China.; 4 Department of Obstetrics and Gynecology, Huzhou Nanxun People's Hospital, Huzhou,313009, China.

**Keywords:** CNKSR2, cervical squamous cell carcinoma, synapse assembly, immune cell infiltration, prognostic value

## Abstract

Connector enhancer of kinase suppressor of Ras 2 (CNKSR2) is a scaffold protein that mediates mitogen-activated protein kinase pathways. However, the molecular function of CNKSR2 in cervical squamous cell carcinoma (CESC) remains unknown. This study aimed to characterize the role of CNKSR2 in patients with CESC. Immunohistochemistry revealed that the expression of CNKSR2 in CESCs is relatively low compared with that in normal cells. We also explored the gene expression profile of high- and low-CNKSR2 expression in patients with cervical cancer. Furthermore, Gene Ontology (GO) and Kyoto Encyclopedia of Genes and Genomes (KEGG) analysis showed that the expression of CNKSR2 was upregulated in synapse assembly, which was coordinately regulated using the cAMP signaling pathway and calcium signaling pathway. The correlation between CNKSR2 and cancer immune cell infiltration was investigated via single-sample gene set enrichment analysis (ssGSEA). High CNKSR2 expression was associated with better overall survival (OS) and disease-free survival (DFS). Interestingly, high CNKSR2 expression was a good predictor of the survival outcome in cervical cancer patients. Additionally, CNKSR2 expression was strongly correlated with diverse immune cells in CESCs, including NK cells and T cells. These findings suggest that CNKSR2 is correlated with prognosis and immune infiltration, laying the foundation for future studies on the functional role of CNKSR2 in CESC.

## Introduction

Cervical squamous cell carcinoma (CESC) is a common malignant gynecologic tumor affecting thousands of women 1. More than 500 000 cases have been diagnosed worldwide 2. CESC often originates from cervical intraepithelial neoplasia and is correlated with human papilloma virus (HPV) infection 3-5. The development of surgical and adjunct treatments has improved the quality of life of CESC patients 6-10, but many patients with CESC are diagnosed with advanced disease due to the technical difficulty of detection, resulting in poor survival outcomes 11,12. Therefore, a new prognostic value in the diagnosis of CESC and new therapeutic targets for improving the treatment outcome must be identified.

Strategies to prevent CESC have been hampered by a limited understanding of the underlying mechanism of this disease. Clinicians have gained valuable experience in the pathological testing of CESC patients by cervical smear cytology, HPV examination and pathological biopsy in colposcopy 13,14. Evidence has confirmed that numerous genes are associated with independent prognostic factors in CESC 15-17. The scaffold protein CNKSR2, also known as MAGUIN and KSR2, is a negative component in tumorigenesis 18,19. Previous studies have demonstrated that CNKSR2 plays a role in some diseases, including breast cancer and thyroid carcinoma 19,20. However, the role of CNKSR2 in CESC remains unknown. Our study first revealed the functional role of CNSKR2 in CESC using The Cancer Genome Atlas (TCGA) and Genotype-Tissue Expression (GTEx) database. At present, we have confirmed the effect of CNKSR2 on CESC, that is, high expression of CNKSR2 was associated with better survival in CESC patients. Enrichment analysis showed that a high-CNKSR2 expression phenotype was correlated with the regulation of synapse assembly. Our results suggest that CNKSR2 serves as an important prognostic marker in CESC and an indicator of immune infiltration.

## Material and methods

### Data Acquisition and Gene Expression Profile Analysis

The gene expression profile and clinical information on CESC were downloaded using UCSC XENA (https://xenabrowser.net/datapages/) from TCGA (https://portal.gdc.cancer.gov/) and the GTEx database. For data processing, we used RNA-Seq data from TCGA and the GTEx database in the TPM format that were uniformly processed using the Toil process 21. First, we analyzed the expression level of CNKSR2 in 304 CESC patients and 13 healthy women. Next, the RNA-Seq gene expression profile and clinical characteristics of 304 CESC patients were retained and further analyzed (Table 1).

### Functional Enrichment Analysis

To elucidate the significant biological process, cellular components and molecular function in the differential CNKSR2 expression groups, Gene Ontology (GO) analysis was performed by R programming of clusterProfiler (3.8.0) 22. The enriched GO terms were selected using an adjusted P-value<0.05 and count>3. Additionally, we performed KEGG analysis to identify CNKSR2-related differentially expressed genes in pathway enrichment analysis with the selection criteria of a false discovery rate (FDR)<0.05.

### Protein-Protein Interaction (PPI) Network Construction

The PPI network was constructed using 673 differentially expressed genes using the STRING database (http://www.string-db.org/) with an interaction score >0.4 23. We used Cytoscape 3.8.0 to visualize the PPI network, and differentially expressed genes were labeled in different colors 24. Subsequently, the CNKSR2-related genes were analyzed using the Molecular Complex Detection (MCODE) plugin to identify the top 10 genes with the strongest interaction in the PPI network. Using topological algorithms including degree, bottleneck, closeness and betweenness, the hub genes in different clusters from the network were further screened using the CytoHubba plugin in Cytoscape 25.

### Immune Cell Infiltration Analysis

Normalized CNKSR2 expression profiles for CESC projects from the TCGA database were collected and compared with different immunocyte signatures using the GSVA package from the R program based on the ssGSEA method 26. ssGSEA classifies different immune cell infiltrations in tumors. Using the signatures of adaptive and innate immune cell types, we quantified the relative tumor infiltration levels of 24 types of immune cell types with published signature lists.

### Survival Analysis and Hazard Model Construction

To evaluate the prognostic value of CNKSR2, we used Cox regression and the Kaplan-Meier method. We calculated the overall survival and disease-specific survival rates of CNKSR2 in CESC patients using the survminer package 27. Multivariate Cox analysis was used to compare the influence of CNKSR2 and other clinicopathologic characteristics—including T stage, N stage, clinical stage, primary therapy outcome, radiation therapy, histological type, menopause status, histologic grade, and presence of keratinizing squamous cell carcinoma—on the survival of CESC patients from TCGA. According to the results of Cox regression, a nomogram model was used to calculate the predictive value of CNKSR2 in estimating the prognosis of CESC patients. We also performed a calibration plot to assess the effectiveness of the model.

### Tissue Samples

A total of 30 CESC patients undergoing cervical cancer radical surgery between 2020 and 2022 in the Affiliated Sir Run Run Shaw Hospital of Zhejiang University School of Medicine were included in this study. This study was approved by the institutional review board of our hospital. We have already got our ethical approval (Approval NO:2023-0153). All methods were performed in accordance with the relevant guidelines and regulations. All patients gave written informed consent.

**Patients' eligibility criteria:** (i) cervical cancer patients and no other underlying diseases; (ii) undergoing cervical cancer radical surgery.

**Patients' exclusion criteria:** (i) other malignant tumors such as breast cancers, ovarian cancer and so on. (ii) abnormal karyotype; (iii) endocrine or metabolic disorders; (iv) autoimmune diseases; (v) neurological diseases; (vi) improper drug treatment, exposure to chemicals or radiation.

### Tissue immunohistochemical staining

Immunohistochemistry was performed using formalin‐fixed paraffin‐embedded histologic sections using anti-CNKSR2 (1:200, Abcam, Ab239026) antibodies. IHC staining was carried out according to the manufacturer's instructions. Then, Color was developed using 3,3′-diaminobenzidine (DAB) for 5 min and histologic sections counterstained with Hematoxylin. Staining was visualized using *a Nikon microscope*.

### Statistical Analysis

R (v.3.6.3) was used to statistically analyze the expression level and other clinical parameters in CESC. The Wilcoxon rank-sum test was used to compare the differential expression of CNKSR2 in CESC samples from TCGA cohorts and the GTEx database. Furthermore, logistic regression was conducted to elucidate the relationship between clinical parameters and CNKSR2. To estimate the predictive value of CNKSR2 in CESC, receiver operating characteristic (ROC) curve analysis was performed, and the area under the curve (AUC) was calculated to determine the authenticity of the testing method.

## Results

### Deleted Expression of CNKSR2 in CESCs

**Figure 1:**
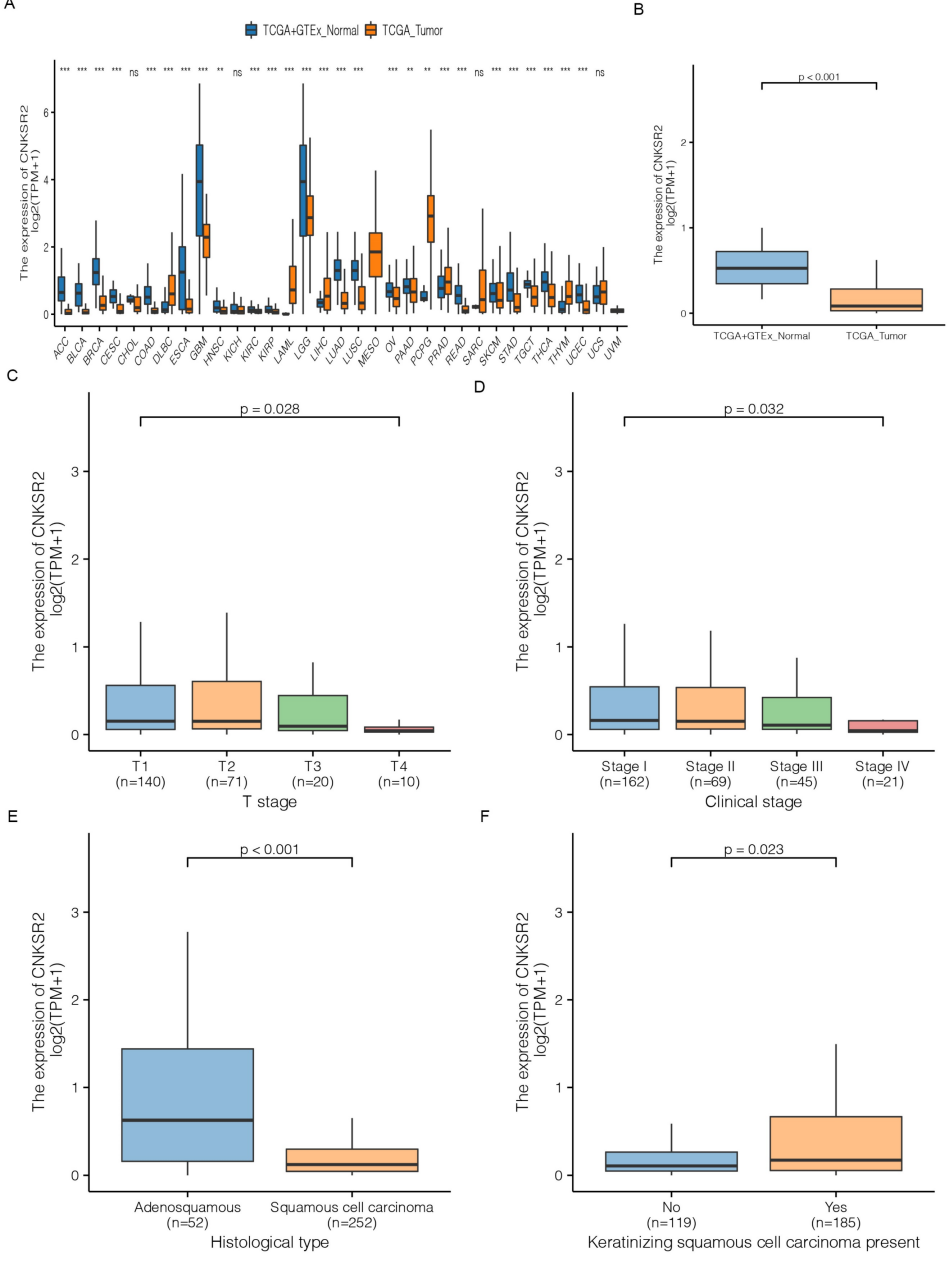
Comparison of CNKSR2 gene expression between cancers and normal tissues in the TCGA and GTEx cohorts. (**A**) Expression level of CNKSR2 in different cancers and normal tissues from the TCGA and GTEx cohorts. ^***^P<0.001, ^**^P<0.01, ns: no sense. (**B**) CNKSR2 expression level in CESC projects from TCGA and normal tissues from TCGA and GTEx cohorts. (**c**) CNKSR2 expression levels in CESC patients with different T stages. (**D**) CNKSR2 expression levels in CESC patients with different clinical stages. (**E**) CNKSR2 expression levels in CESC patients with different histologic types. (**F**) CNKSR2 expression levels in CESC patients with the presence of different keratinizing squamous cell carcinomas. TCGA, The Cancer Genome Atlas.

We initially evaluated the transcriptional level of CNKSR2 in multiple tumors from TCGA and the GTEx database. Analysis of multiple cancers revealed that compared to adjacent normal tissues, the mRNA expression of CNKSR2 was significantly lower in cancers—such as ovarian serous cystadenocarcinoma (OV), pancreatic cancer (PAAD), pheochromocytoma and paraganglioma (PCPG), prostate cancer (PRAD), rectal cancer (READ), skin melanoma (SKCM), gastric cancer (STAD), testicular cancer (TGCT), thyroid cancer (THCA), thymic cancer (THYM) and endometrial carcinoma (UCEC) (Figure 1A). Data from TCGA and the GTEx database indicated that CNKSR2 was extremely downregulated in CESCs in terms of mRNA expression (Figure 1B). The characteristics of CESC patients are listed in Table 1.

Further subgroup analysis of multiple clinicopathological features of CESC samples showed that the deleted transcriptional levels was consistent with previous findings. The expression of CNKSR2 was significantly lower in CESC patients than in normal controls in subgroups based on T stage, clinical stage, histological type and keratinizing squamous cell carcinoma (Figure 1C-F). Additionally, high CNKSR2 expression was found in CESC patients with histological type (P<0.001) and keratinizing squamous cell carcinoma (P = 0.026) according to logistic analysis (Table 2). Therefore, CNKSR2 expression may play a prominent role in CESC patients.

### Enrichment Analysis of CNKSR2 Co-expressed Genes in CESC

**Figure 2:**
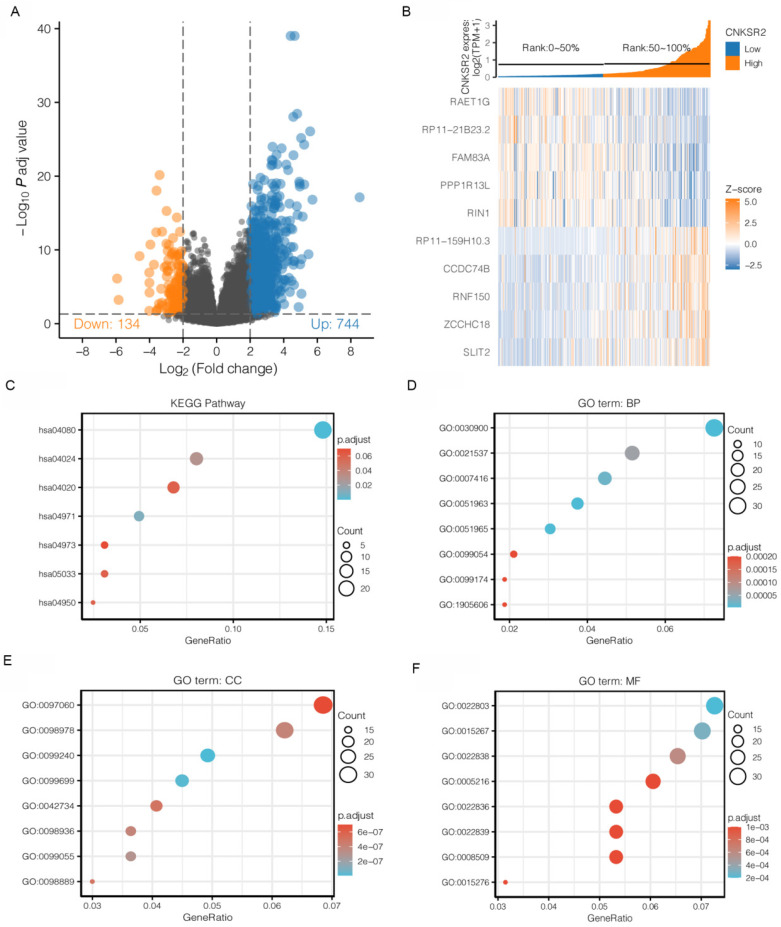
Differentially expressed genes in the high- and low-CNKSR2 expression groups in the CESC cohorts. (**A**) Differentially expressed genes between different CNKSR2 expression groups. (**B**) CNKSR2-correlated gene expression in CESC cohorts. (**C**) KEGG pathway enrichment analysis of CNKSR2 coexpression genes. (**D**) Biological process terms of CNKSR2 coexpression genes. (**E**) Cellular component terms of CNKSR2 coexpression genes. (**F**) Molecular function terms of CNKSR2 coexpression genes.

To identify the CNKSR2-related biological process in CESC, we evaluated the CNKSR2 co-expression mode in the CESC cohort from TCGA. 744 genes (orange dots) were positively correlated with CNKSR2, and 134 genes were negatively correlated with CNKSR2 (Figure 2A). The top 10 significant genes which positively and negatively associated with CNKSR2 were shown in the heat map (Figure 2B). CNKSR2 had a strong positive correlation with the expression of RP11-159H10.3 (r=0.50; P=1.09E-20), CCDC74B (r=0.49; P=1.02E-19), RNF150 (r=0.49; P=1.63E-19), ZCCHC18 (r=0.49; P=2.40E-19), and SLIT2 (r=0.48; P=7.69E-19).

GO terms and KEGG pathway analysis using the clusterProfiler package revealed that the cAMP signaling pathway and calcium signaling pathway were enriched (Figure 2C and Table 3). Additionally, GO analysis showed that CNKSR2 co-expressed genes primarily participate in the regulation of synapse assembly and are particularly expressed in synaptic membranes, glutamatergic synapses, intrinsic components of synaptic membranes, integral components of synaptic membranes and presynaptic membranes (Figure 2D-F). These results suggest the widespread impact of different expression levels of CNKSR2.

### Gene Co-Occurrence of CNKSR2 Alteration in CESC

**Figure 3:**
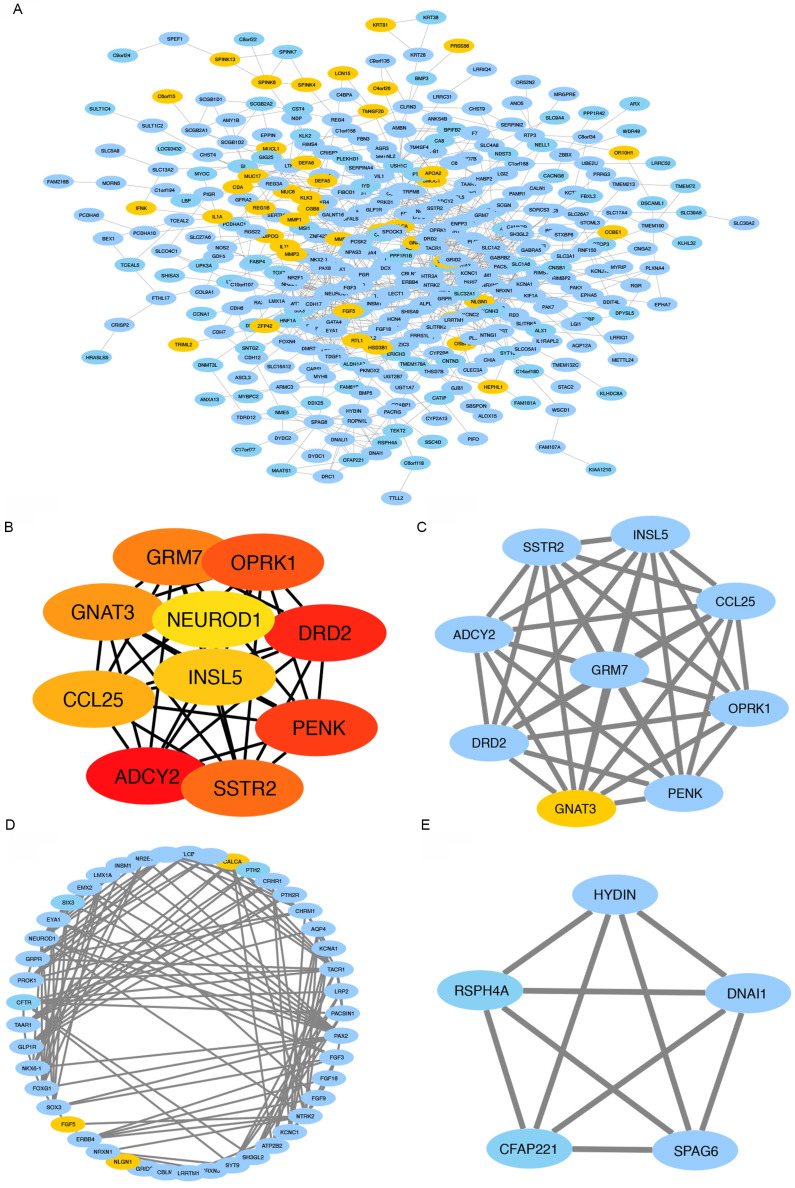
CNKSR2 co-occurrence profiles in CESC. (**A**) Network of co-occurrence genes along with CNKSR2. (**B**) Top 10 CNKSR2-related genes in the network. (**C**)-(**E**) Three clusters with high interaction degrees were calculated using CytoHubba.

To further explore the gene co-occurrence of CNKSR2 in CESC, we analyzed the functional relationships of genetic risk factors (Figure 3A). The frequently correlated genes were dopamine receptor D2 (DRD2; degree=19), adenylate cyclase 2 (ADCY2; degree=18), somatostatin receptor 2 (SSTR2; degree=12), proenkephalin (PENK; degree=21), opioid receptor kappa 1 (OPRK1; degree = 16), glutamate metabotropic receptor 7 (GRM7; degree=13), G protein subunit alpha transducin 3 (GNAT3; degree=14), C-C motif chemokine ligand 25 (CCL25; degree=11), insulin-like 5 (INSL5; degree=9), and neuronal differentiation 1 (NEUROD1; degree=22) (Figure 3B). Three clusters had significant co-occurrence with CNKSR2 (Figure 3C-E).

### Validating expression and clinical prognostic value by immunohistochemical

**Figure 4:**
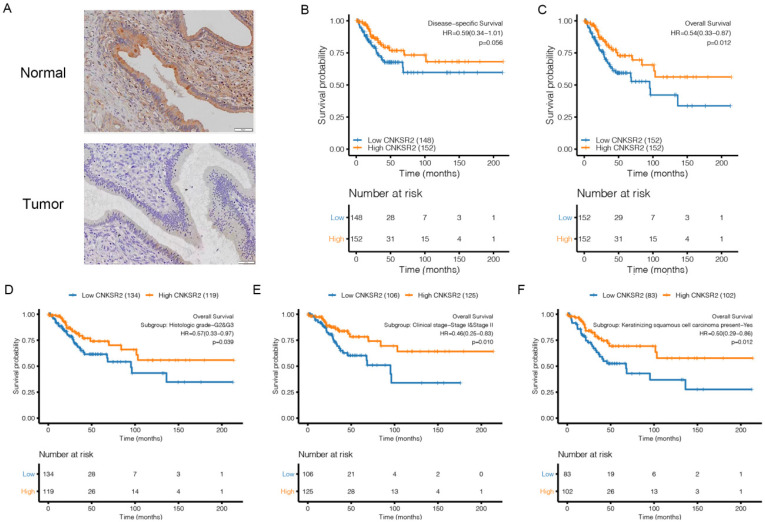
(**A**) CLDN10 expression in PTC tissues and adjacent normal tissues assayed by IHC. The scales bar to indicate 50 μm. (**B**)-(**C**) Characteristics of the clinical parameters and CNKSR2 expression in CESC patients from the TCGA cohorts. Kaplan-Meier plot regarding disease-specific survival (**B**) and overall survival (**C**) in the high- and low-CNKSR2 expression groups from the CESC cohorts. (**D**)-(**F**) Subgroup survival analysis in G2 and G3 (**D**), Stage I and Stage II (**E**) and keratinizing squamous cell carcinoma (**F**) groups in high- and low-CNKSR2 groups from the CESC cohorts.

To determine the role of CNKSR2 in the clinical progression of CESC, we performed immunohistochemical analysis on the 30 CESC tissue and normal adjacent tissues samples. Our results showed that CNKSR2 levels are elevated in normal adjacent tissues compared to CESC tissue (Figure 4A).Then we compared the differences in the survival rates considering the stage and CNKSR2 expression by log-rank analysis in Kaplan-Meier curves. We found that the disease-specific survival rate (HR=0.59; 95% CI: 0.34-1.01) was higher in patients with high CNKSR2 expression than in those with low CNKSR2 expression in TCGA (Figure 4B). Additionally, patients with high CNKSR2 expression had a higher overall survival rate than those with low CNKSR2 expression in TCGA (HR=0.54; 95% CI: 0.33-0.87; P=0.012; Figure 4C). The survival rate was higher in patients with high CNKSR2 expression than in those with low CNKSR2 expression in the histologic grade (G2&G3) group in the CESC project of the TCGA cohort (HR: 0.57; 95% CI: 0.33-0.97; P=0.039; Figure 4D). Additionally, the survival rates were higher in patients with high CNKSR2 expression in the clinical stage (Stage I & Stage II) group (HR: 0.46; 95% CI: 0.25-0.83; P=0.010) (Figure 4E) and CESC patients with keratinizing squamous cell carcinoma (HR=0.50; 95% CI: 0.29-0.86; P=0.012; Figure 4f).

### CNKSR2 Expression is Associated with Survival Outcome

**Figure 5:**
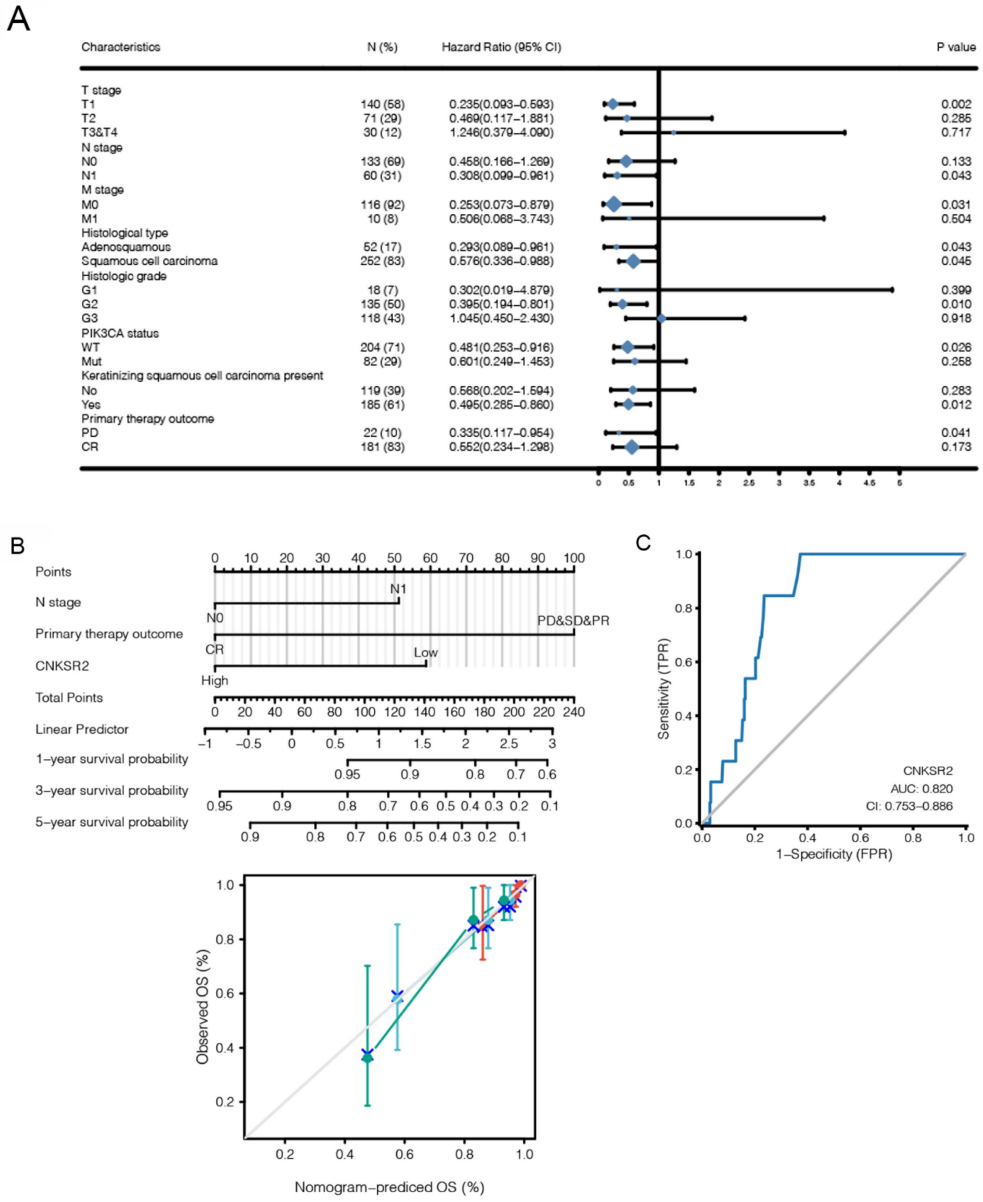
(**A**) Multivariate analysis of the CESC project in TCGA. (**B**) Nomogram model regarding the N stage, primary therapy outcome and CNKSR2 gene expression in the TCGA cohorts. (**C**) Receiver operating characteristic (ROC) curves regarding CNKSR2 gene expression in TCGA.

CNKSR2 was also identified as a possible prognostic biomarker using a multivariate hazard model (Figure 5A). CNKSR2 had prognostic significance in TCGA (Table 4; HR: 0.302; 95% CI: 0.115-0.792; P=0.015). Based on multivariate analysis, we applied N stage, primary therapy outcome and CNKSR2 expression to our nomogram model, which indicated that CNKSR2 has prognostic value in CESC (Figure 5B). Regarding the effectiveness of CNKSR2 as a biomarker in CESC, we examined the AUC values in the ROC curves for TCGA (Figure 5C). ROC analysis revealed that CNKSR2 had stable predictive values in CESC patients (AUC=0.820; 95% CI: 0.753-0.886).

### Immune Cell Infiltration in CESCs with Different CNKSR2 Expression

**Figure 6:**
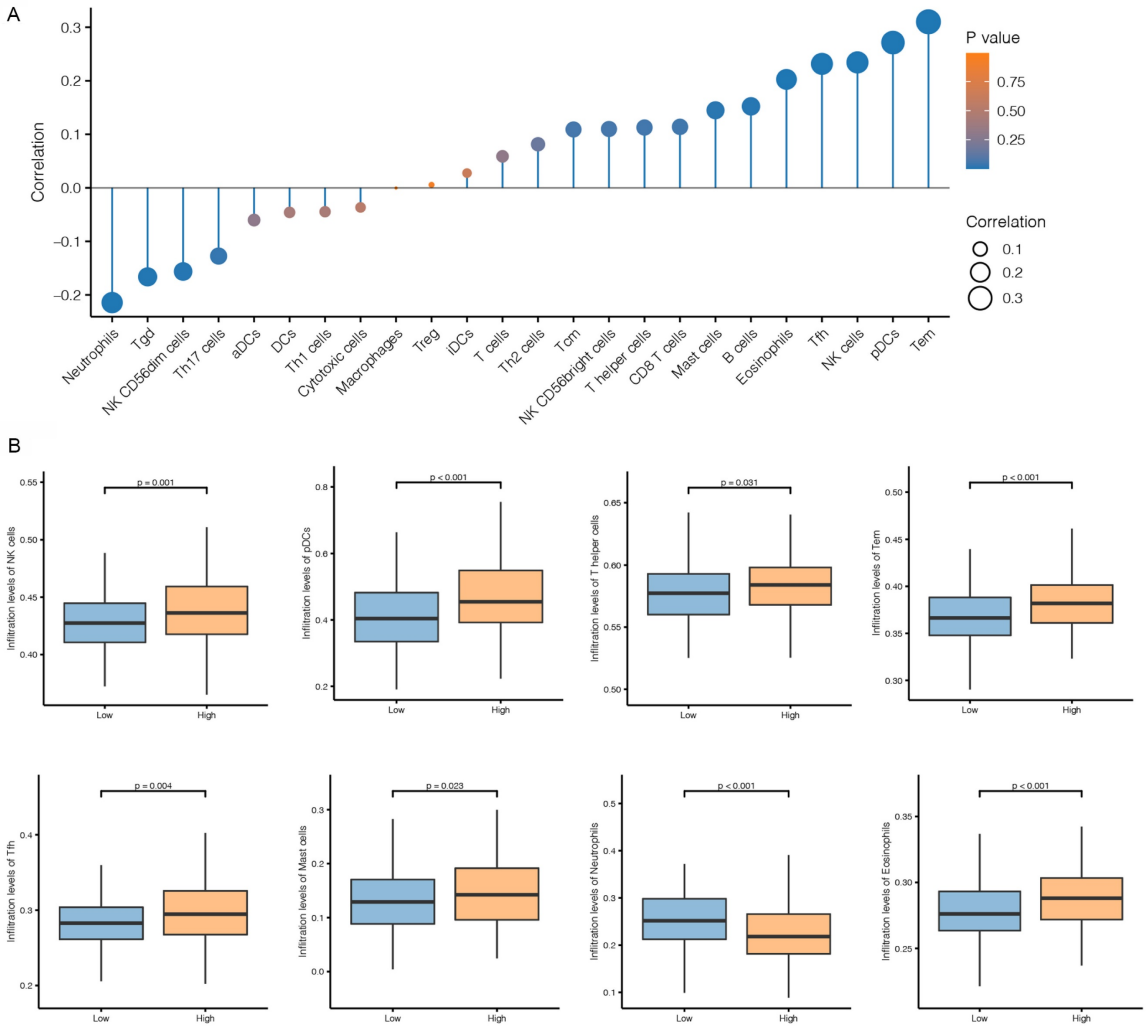
Immune cells in CESC tissues derived from the TCGA cohorts. (**A**) Correlation of different immune cells in the low- and high-CNKSR2 expression CESC cohorts in the TCGA database. (**B**) Comparison of different immune cells in high- and low-CNKSR2 expression groups in the CESC cohorts in the TCGA database.

To explore the difference in the immune environment in CESC patients with differential expression of CNKSR2 in the TCGA cohort, the data were analyzed. Different immune cell infiltration occurred in CESC patients with differential CNKSR2 expression (Figure 6A). The number of NK cells, dendritic cell leakage, T cells, mast cells and eosinophils in the high-CNKSR2 expression cohort were all higher than those in the low-CNKSR2 expression cohort (Figure 6B).

## Discussion

CNKSR2 is a scaffold gene that participates primarily in signal transduction and is a protein that mediates the mitogen-activated protein kinase pathway downstream of Ras. CNKSR2 is associated with different hereditary diseases, including nonsyndromic X-linked intellectual disability, mental retardation and undetermined early-onset epileptic encephalopathy. In the present study, we first revealed that CNKSR2 was correlated with CESC tumor tissue. Additionally, a similar association was found in multiple cancers from TCGA.

Previous studies have revealed that the Smurf2 E3 ubiquitin ligase may induce CNKSR2 in cancer cells and participate in cancer cell proliferation 18. We performed an enrichment analysis to explore the CNKSR2 coexpression genes in cervical cancer progression. CNKSR2 coexpression genes participate primarily in regulating synapse assembly and may couple signal transduction to membrane or cytoskeletal remodeling. During tumorigenesis and progression, families of immunosuppressive molecules inhibit the activation of tumor reactive T cell receptors through immune synapse isolation 28. Additionally, NK cells activate T cells by activating dendritic cell precursors to generate immune synapses 29. For example, BTN3A1 inhibits the activation of tumor-killing αβ T cells and γδ T cells by preventing the N-glycosylation process in tumor development. Thus, the development of CD277-specific antibodies led to a new therapeutic strategy for tumor chemotherapy resistance by targeting BTN3A1 in cancer therapy 28. Notably, the efficacy of CAR-modified immune cells (including CAR-T and CAR-NK cells) in tumor immunotherapy can also be determined by the quality of immune synapses 30. Various immune cells infiltrated cervical cancer with high CNKSR2 expression. Therefore, we infer that CNKSR2 may participate in the formation of synapses between immune cells in the process of tumor immunity, promoting the function of tumor immunity and improving the survival outcome of cervical cancer.

CNKSR2 is involved mainly in synaptic assembly in the high-CNKSR2 expression group through enrichment analysis. High synapse quality can be used as a marker of a good prognosis. Similarly, we assessed CNKSR2 using various prognostic models and survival curves to evaluate the prognostic value of CNKSR2 and found that CNSKR2 has excellent effectiveness as an independent prognostic indicator. CNKSR2 is also critical in the application of distinguishing cervical cancer from healthy women in tumor prediction models in ROC tests (AUC=0.820). Therefore, we hypothesized that CNKSR2 might mediate signal transduction in tumors through synaptic aggregation, thereby inhibiting tumor development. CNKSR2 expression in patients with thyroid papillary carcinoma is often increased, particularly in *in vitro* experiments revealing that the knockdown of CNKSR2 inhibits ERK phosphorylation and Notch signaling transduction decreases, resulting in damaged tumor proliferation 19. Furthermore, the E3 ubiquitin ligase SMUF2 inhibits the invasion and migration of breast cancer mainly in a scaffold protein CNKSR2-dependent manner 18, also suggesting that CNKSR2, as a scaffold protein, participates in RAS-dependent signaling pathways, highlighting that CNSR2 not only is involved in the signal transduction of kinases but also affects the function of ubiquitin ligases. Thus, the pathogenesis and mechanism of CNKSR2 in cervical cancer warrant further exploration.

Additionally, considering the infiltration of immune cells, cervical cancer patients with high CNKSR2 expression can increase the possibility of multiple immune cell infiltrations in the tumor environment. We found that the proportion of many immune cells involved in tumor immunity, such as NK cells, T cells and dendritic cells, were significantly involved in antigen presentation. Interestingly, among the immune cells that infiltrated cervical cancer, the increased proportion of activated memory CD4+ T cells has a good survival prognosis, and vice versa. Additionally, the combination of NK cells, activated CD4+ T cells and activated mast cells can improve the ability to predict the overall survival of patients with cervical cancer 31. Presently, various chemotherapeutic drugs for gynecological malignant tumors have been suggested to improve the antibody-dependent cell-mediated cytotoxicity (ADCC) function of NK cells and exert their antitumor ability, such as rituximab, herceptin, disialoganglioside (GD2), cetuximab and panitumumab^32^. However, the interrelationship among NK cells, mast cells and T cells in the immune microenvironment of cervical cancer remains a topic worth exploring. Additionally, the effects of high CNKSR2 expression and increased numbers of NK cells, mast cells and T cells on synaptic formation between different immune cells and tumor immune escape in cervical cancer have not been studied, warranting further study.

## Conclusion

Our study confirmed that CNKSR2 participates in synaptic assembly, particularly in cervical cancer with high CNKSR2 expression in various infiltrating immune cells, and can be used as a good tool for the prognosis of cervical cancer patients. Furthermore, the cAMP signaling pathway and calcium signaling pathways, which were reported to play a role in cervical cancer, were also enriched in cervical cancer tissues with high CNKSR2 expression. However, we only used bioinformatics algorithms to estimate the biological role of CNKSR2 in cervical cancer, and we could not directly observe the biological function of CNKSR2 in cervical cancer. Further experimental verification should be performed to observe the biological effects of CNKSR2 on cervical cancer.

## Figures and Tables

**Table 1:** Baseline characteristics of CESC patients in TCGA

Characteristic	Level	Patients (n=304)
Age(years)		46.00 (38.00,56.25)
Average Height(cm)		160.00 (157.00,165.00)
Average Weight(kg)		70.00 (58.00,83.00)
T stage (n/%)	T1	140 (58.1%)
	T2	71 (29.5%)
	T3	20 (8.3%)
	T4	10 (4.1%)
N stage (n/%)	N0	133 (68.9%)
	N1	60 (31.1%)
M stage (n/%)	M0	116 (92.1%)
	M1	10 (7.9%)
Clinical stage (n/%)	Stage I	162 (54.5%)
	Stage II	69 (23.2%)
	Stage III	45 (15.2%)
	Stage IV	21 (7.1%)
Radiation therapy (n/%)	No	122 (40.1%)
	Yes	182 (59.9%)
Primary therapy outcome (n/%)	CR	181 (83.4%)
	PD	22 (10.1%)
	PR	8 (3.7%)
	SD	6 (2.8%)
Race (n/%)	Asian	20 (7.7%)
	Black or African American	30 (11.6%)
	White	209 (80.7%)
Histological type (n/%)	Adenosquamous	52 (17.1%)
	Squamous cell carcinoma	252 (82.9%)
Histologic grade (n/%)	G1	18 (6.6%)
	G2	135 (49.6%)
	G3	118 (43.4%)
	G4	1 (0.4%)
Menopause status (n/%)	Peri	25 (10.8%)
	Post	82 (35.5%)
	Pre	124 (53.7%)
Birth control pill history (n/%)	No	89 (56.7%)
	Yes	68 (43.3%)
Presence of keratinizing squamous cell carcinoma (n/%)	No	119 (39.1%)
	Yes	185 (60.9%)
Smoker (n/%)	No	144 (55.2%)
	Yes	117 (44.8%)
PIK3CA status (n/%)	Mut	82 (28.7%)
	WT	204 (71.3%)

**Table 2:** Logistic regression of CESC patients with different CNKSR2 expression levels

Characteristic	Odds Ratio (OR)	P value
T stage (T2 & T3 & T4 vs. T1)	0.70 (0.42-1.18)	0.181
N stage (N1 vs. N0)	0.79 (0.43-1.46)	0.455
M stage (M1 vs. M0)	1.07 (0.28-4.04)	0.917
Clinical stage (Stage II & Stage III & Stage IV vs. Stage I)	0.64 (0.40-1.01)	0.057
Primary therapy outcome (CR vs. PD & SD & PR)	2.04 (0.99-4.39)	0.059
Histological type (squamous cell carcinoma vs. adenosquamous)	0.21 (0.10-0.42)	<0.001
Histologic grade (G3 & G4 vs. G1 & G2)	1.02 (0.63-1.65)	0.927
Presence of keratinizing squamous cell carcinoma (Yes vs. No)	1.70 (1.07-2.71)	0.026
PIK3CA status (Mut vs. WT)	1.60 (0.96-2.70)	0.074

**Table 3:** Enrichment analysis of CNKSR2-correlated genes

Term	ID	Description	Counts
KEGG pathways	hsa04080	neuroactive ligand-receptor interaction	24
	hsa04971	gastric acid secretion	8
	hsa04024	cAMP signaling pathway	13
	hsa05033	nicotine addiction	5
	hsa04950	maturity onset diabetes of the young	4
	hsa04020	calcium signaling pathway	11
	hsa04973	carbohydrate digestion and absorption	5
BP terms	GO:0030900	forebrain development	31
	GO:0051963	regulation of synapse assembly	16
	GO:0051965	positive regulation of synapse assembly	13
	GO:0007416	synapse assembly	19
	GO:0021537	telencephalon development	22
	GO:1905606	regulation of presynapse assembly	8
	GO:0099174	regulation of presynapse organization	8
	GO:0099054	presynapse assembly	9
CC terms	GO:0097060	synaptic membrane	32
	GO:0099056	integral component of presynaptic membrane	12
	GO:0045211	postsynaptic membrane	25
	GO:0098793	presynapse	32
	GO:0099634	postsynaptic specialization membrane	13
	GO:0099060	integral component of postsynaptic specialization membrane	11
	GO:0098948	intrinsic component of postsynaptic specialization membrane	11
	GO:0098982	GABA-ergic synapse	10
MF terms	GO:0022803	passive transmembrane transporter activity	30
	GO:0015267	channel activity	29
	GO:0022838	substrate-specific channel activity	27
	GO:0008509	anion transmembrane transporter activity	22
	GO:0022839	ion gated channel activity	22
	GO:0005216	ion channel activity	25
	GO:0022836	gated channel activity	22
	GO:0015276	ligand-gated ion channel activity	13

**Table 4:** Prognostic value of CNKSR2 and other clinical parameters in the TCGA cohorts in the multivariate hazard model

	Univariate analysis		Multivariate analysis	
Characteristic	HR (95% CI)	P value	HR (95% CI)	P value
T stage (T2 & T3 & T4 vs. T1)	1.846 (1.045-3.260)	0.035	1.203 (0.455-3.185)	0.709
N stage (N1 vs. N0)	2.695 (1.358-5.349)	0.005	2.660 (1.082-6.541)	0.033
Clinical stage (Stage II & Stage III & Stage IV vs. Stage I)	1.429 (0.896-2.280)	0.134		
Primary therapy outcome (CR vs. PD & SD & PR)	0.074 (0.040-0.138)	<0.001	0.171 (0.062-0.470)	<0.001
Radiation therapy (Yes vs. No)	1.153 (0.681-1.951)	0.596		
Histological type (squamous cell carcinoma vs. adenosquamous)	1.010 (0.530-1.926)	0.976		
Menopause status (post vs. pre & peri)	1.275 (0.744-2.185)	0.376		
Histologic grade (G3 & G4 vs. G1 & G2)	0.889 (0.527-1.502)	0.661		
Smoker (Yes vs. No)	1.470 (0.900-2.401)	0.124		
Birth control pill history (Yes vs. No)	0.677 (0.326-1.404)	0.294		
Keratinizing squamous cell carcinoma present (Yes vs. No)	1.395 (0.813-2.394)	0.227		
Age (>50 vs. ≤50)	1.317 (0.825-2.101)	0.248		
Height (>160 vs. ≤160)	1.092 (0.633-1.883)	0.752		
Weight (>70 vs. ≤70)	0.736 (0.446-1.214)	0.23		
Race (Asian & Black or African American vs. White)	0.841 (0.427-1.658)	0.618		
PIK3CA status (Mut vs. WT)	1.011 (0.599-1.707)	0.967		
CNKSR2 (High vs. Low)	0.536 (0.330-0.869)	0.012	0.302 (0.115-0.792)	0.015
